# Supplementary material for: HIV-1 uncoating location dictates sites of integration
Source: Nat Commun. 2026 Apr 9;17:4840. doi: 10.1038/s41467-026-71679-3 (PMC13223229; doi:10.1038/s41467-026-71679-3)
Supplement: Supplementary file 1 — Supplementary Information [file 41467_2026_71679_MOESM1_ESM.pdf]

Supplementary Information:

HIV-1 uncoating location dictates sites of integration

Ryan C. Burdick[1,*], Sean C. Patro[2], Ellie Bare[1], Rokeya Siddiqui[1], Krista A. Delviks-Frankenberry[1], Olga A. Nikolaitchik[3], Stephen H. Hughes[4], Xiaolin Wu[2], Wei-Shau Hu[3], and Vinay K. Pathak[1,*]

[1]Viral Mutation Section, HIV Dynamics and Replication Program, Center for Cancer Research, National Cancer Institute, National Institutes of Health, Frederick, MD, USA

[2]Cancer Research Technology Program, Frederick National Laboratory for Cancer Research, Frederick, MD, USA

[3]Viral Recombination Section, HIV Dynamics and Replication Program, Center for Cancer Research, National Cancer Institute, National Institutes of Health, Frederick, MD, USA

[4]HIV Dynamics and Replication Program, Center for Cancer Research, National Cancer Institute, National Institutes of Health, Frederick, MD, USA

Correspondence to: Email: burdickrc@mail.nih.gov and pathakv@mail.nih.gov

This pdf file includes:

Supplementary Figures 1-4

Supplemental Tables 1-5

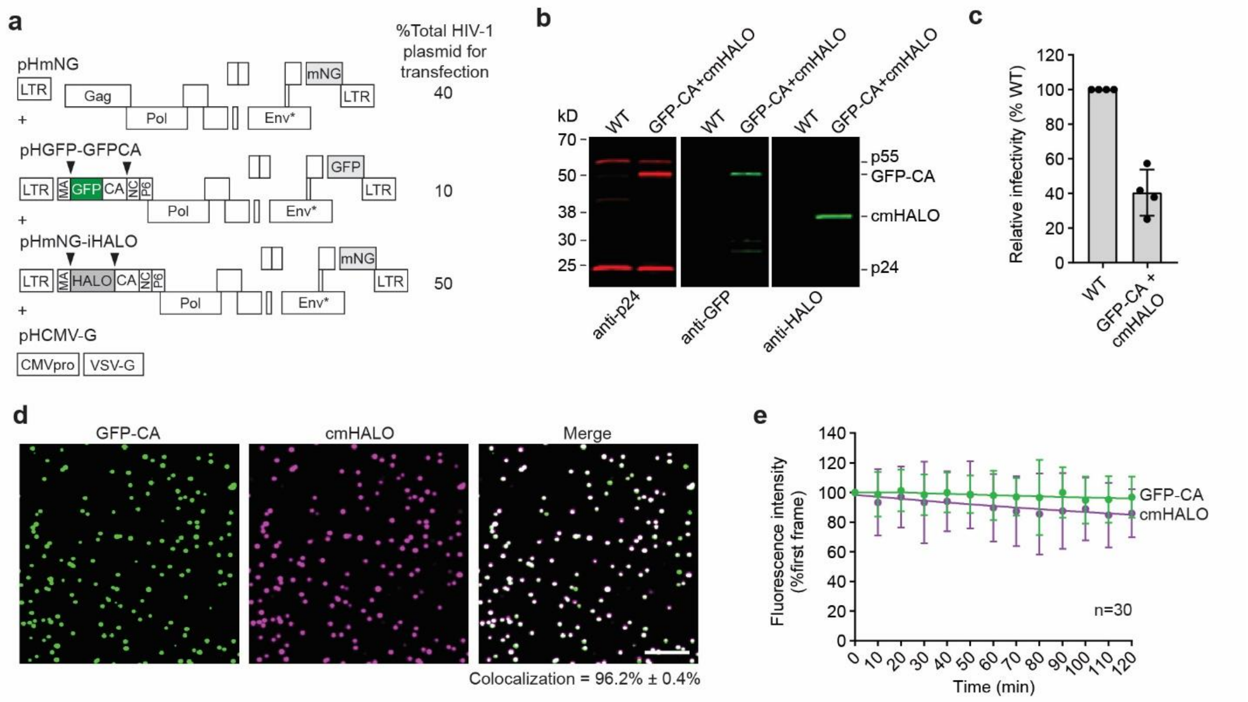

Supplementary Fig. 1. Live-cell imaging assay to visualize nuclear speckles and HIV-1 uncoating. a, HIV-1 plasmid design for virion labeling. HIV-1 plasmids expressing WT Gag (pHmNG) or Gag with internal GFP (pHGFP-GFPCA) or internal HALO (pHmNG-iHALO) were transfected at 40%, 10%, and 50%, respectively, of the total plasmid amount. Black triangles mark protease cleavage sites. Proteolytic cleavage of Gag from pHGFP-GFPCA generates GFP-CA upon virus maturation, while cleavage of Gag from pHmNG-iHALO produces fully processed HALO protein. Asterisk denotes a mutation in the Env that generates a premature stop codon; virions were pseudotyped with VSV-G. The GFP and mNG reporters in the *nef* open reading frame are expressed in virus-producing cells but not incorporated into virions. **b,** Western blot analysis of viral lysates comparing WT virions (generated using only pHmNG) and dual-labeled virions (generated using a combination of pHmNG, pHGFP-GFP-CA, and pHmNG-iHALO as described in **a**). **c,** Effect of labeling on virus infectivity. TZM-bl cells were infected with p24 CA-normalized amounts of WT or dual-labeled virus. Luciferase activity was measured 48 hours post-infection (n = 4 independent experiments). Error bars represent standard deviation. **d,** Representative images of dual-labeled virions. Virions containing GFP-CA and cmHALO were centrifuged onto a chambered slide and imaged by confocal microscopy. The percentage of GFP-CA spots that colocalize with cmHALO is shown (average ± standard deviation from 4 different virus preparations). Scale bar, 5 µm. **e,** Quantification of 30 randomly selected viral cores that did not

uncoat during the 2-hour long movies described in **Fig. 1**. Fluorescence intensities of the GFP-CA and cmHALO signals were normalized to the first frame and curve fitting was applied. Error bars represent standard deviation. Source data are provided as a Source Data file.

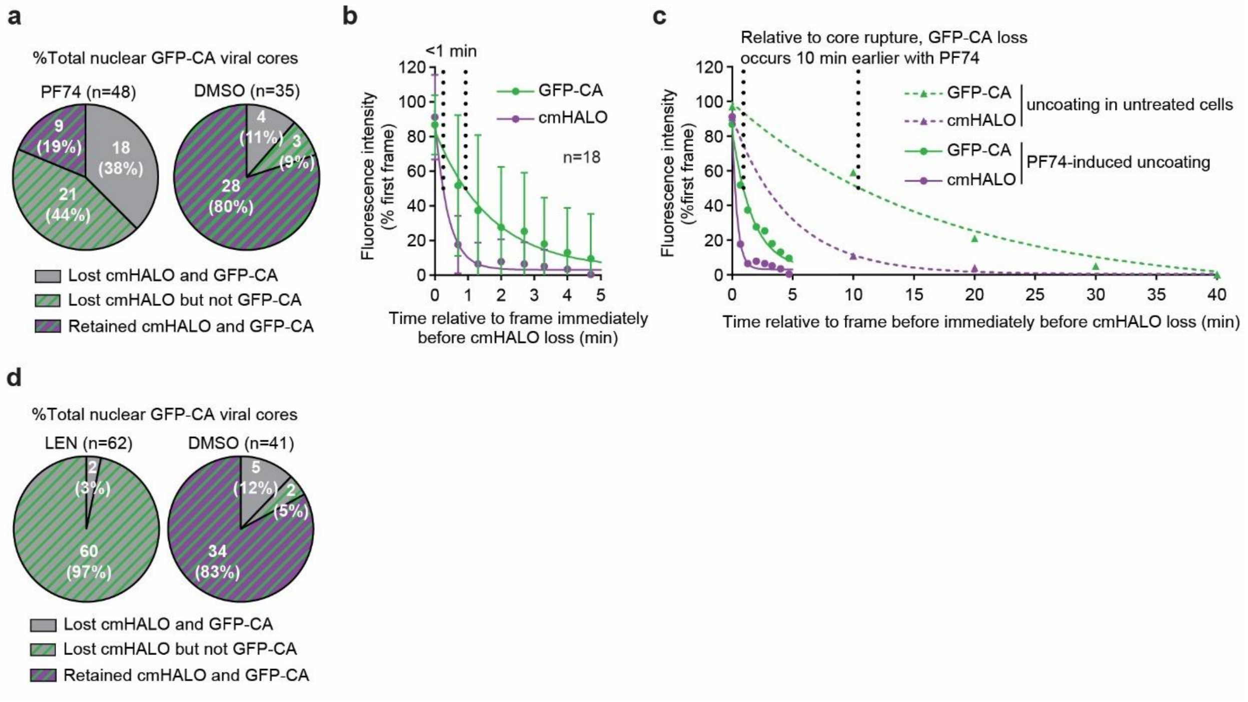

Supplementary Fig. 2. Characterization of cmHALO and GFP-CA loss following treatment with PF74 or LEN. a, Percentage of nuclear GFP-CA–labeled viral cores that retained cmHALO, lost cmHALO but not GFP-CA, or lost both cmHALO and GFP-CA following treatment with PF74 (n = 48 viral cores) or DMSO (control; n = 35 viral cores). **b**, Quantification of fluorescence intensities for the 18 viral cores that lost both cmHALO and GFP-CA following PF74 treatment. Fluorescence intensities of the GFP-CA and cmHALO signals were normalized to the first frame and are plotted relative to the frame immediately before initial cmHALO signal loss. Curve fitting was applied to determine the time at which ~50% signal loss occurred for each fluorophore. Error bars represent standard deviation. **c**, Comparison of the kinetics of cmHALO and GFP-CA loss in untreated cells (same as **Fig. 1e**) and in cells treated with PF74 (same as **b**). Standard deviation bars were omitted for clarity. **d**, Same as **a**, except following treatment with LEN (n = 62 viral cores) or DMSO (n = 41 viral cores). For **a** and **d**, numbers are shown, with percentages in parentheses. Source data are provided as a Source Data file.

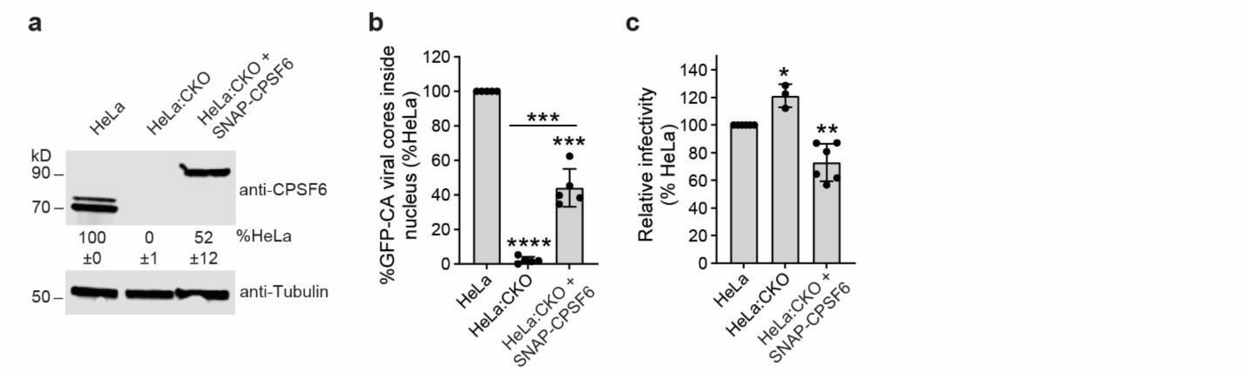

Supplementary Fig. 3. Analysis of nuclear import and infectivity in a HeLa-based cell line in which endogenous CPSF6 was knocked out and SNAP-CPSF6 was expressed from a retroviral vector. a, Western blot of parental HeLa cells, CPSF6 knockout cells (HeLa:CKO), and HeLa:CKO cells expressing SNAP-tagged CPSF6. Numbers below blot indicate CPSF6 levels relative to HeLa (average ± standard deviation from 3 blots). **b,** Nuclear import efficiency of GFP-CA-labeled viral cores in parental HeLa cells, HeLa:CKO, and HeLa:CKO cells expressing SNAP-tagged CPSF6. The percentage of total GFP-CA puncta localized inside the nucleus at 6 hours post-infection was determined and normalized to parental HeLa cells (n = 5 independent experiments). **c,** Relative infectivity of a GFP-reporter virus in the indicated cell lines (n = 3-5 independent experiments). Cells were initially gated based on FSC-A and SSC-A. Singlets were identified by FSC-A versus FSC-H gating. A GFP-positive gate was established using uninfected cells. The percentage of GFP^+ cells was then quantified. In **b** and **c,** statistical significance was determined using two-sided Welch's t-tests. ****$p < 0.0001$, ***$p < 0.001$, **$p < 0.01$, *$p < 0.05$. Error bars represent standard deviation. Source data are provided as a Source Data file.

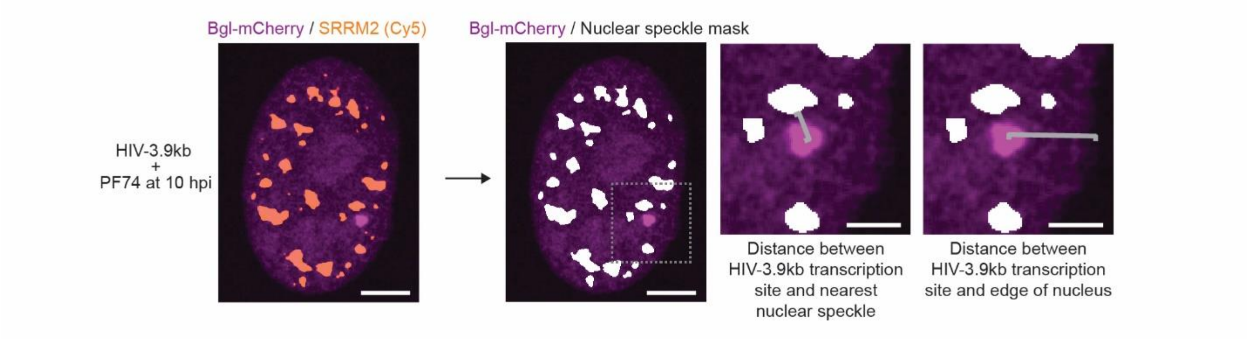

Supplementary Fig. 4. Assay to quantify nuclear localization of transcriptionally active proviruses. Representative example of a transcriptionally active provirus derived from PF74-induced uncoating of viral core containing HIV-3.9kb, shown in relation to NSs (same as in **Fig. 5c**). A NS mask was generated using SRRM2 signal to determine provirus colocalization with NSs and to measure the distance between the provirus and nearest NS edge. Distance from the provirus to the nuclear periphery was calculated based on the boundary defined by a sharp drop in the diffuse Bgl-mCherry signal, which was previously shown to align with the nuclear envelope. Scale bars, 5 µm; insets, 2 µm. Source data are provided as a Source Data file.

Supplemental Table 1. Integration Site Analysis

Sample	Cell type	Treatment	Unique sites	In RefSeq[3] genes (% Total)	Avg. gene density ± 500 kb	In SPADs (% Total)	In LADs (% Total)
HIV-8.4kb	HeLa	-	17,249	77.4	19.7	29.9	7.7
HIV-8.4kb	HeLa	10 µM PF74 at 8 hpi	20,688	73.4	15.2	19.6	12.1
HIV-3.1kb	HeLa	10 µM PF74 at 10 hpi	6,149	69.2	8.5	4.2	16.0
HIV-3.1kb	HeLa	100 nM LEN at 10 hpi	8,070	67.6	11.7	11.4	15.7
HIV-3.1kb	HeLa	10 nM LEN at 10 hpi	8,566	68.6	12.4	13.0	14.7
HIV-3.1kb	HeLa	-	3,554	73.6	18.0	27.1	10.0
NL4-3[1]	PBMC	-	385,205	81.9	19.4	28.5	7.1
RIC[2]	-	-	259,649	38.9	7.2	4.3	39.8

[1]Integration sites from previously reported HIV-1-infected human PBMCs[59].

[2] Random integration control (RIC). An *in silico* dataset simulation of random integration sites across the human genome (hg19) were reported previously[59].

[3]Well-annotated genes in the National Center for Biotechnology Information (NCBI) Reference Sequence database.

Supplemental Table 2. *P* values: Integration into RefSeq genes[1]

	HIV-8.4kb HeLa	HIV-8.4kb HeLa 10 µM PF74 at 8 hpi	HIV-3.1kb HeLa 10 µM PF74 at 10 hpi	HIV-3.1kb HeLa 100 nM LEN at 10 hpi	HIV-3.1kb HeLa 10 nM LEN at 10 hpi	HIV-3.1kb HeLa	NL4-3 PBMC[2]	RIC[3]
HIV-8.4kb HeLa	-	$<1\times10^{-15}$	$<1\times10^{-15}$	$<1\times10^{-15}$	$<1\times10^{-15}$	1.27×10^{-6}	$<1\times10^{-15}$	$<1\times10^{-15}$
HIV-8.4kb HeLa 10 µM PF74 at 8 hpi	-	-	1.69×10^{-10}	$<1\times10^{-15}$	$<1\times10^{-15}$	7.58×10^{-1}	$<1\times10^{-15}$	$<1\times10^{-15}$
HIV-3.1kb HeLa 10 µM PF74 at 10 hpi	-	-	-	5.38×10^{-2}	5.04×10^{-1}	3.56×10^{-6}	$<1\times10^{-15}$	$<1\times10^{-15}$
HIV-3.1kb HeLa 100 nM LEN at 10 hpi	-	-	-	-	1.67×10^{-1}	9.56×10^{-11}	$<1\times10^{-15}$	$<1\times10^{-15}$
HIV-3.1kb HeLa 10 nM LEN at 10 hpi	-	-	-	-	-	5.02×10^{-8}	$<1\times10^{-15}$	$<1\times10^{-15}$
HIV-3.1kb HeLa	-	-	-	-	-	-	$<1\times10^{-15}$	$<1\times10^{-15}$
NL4-3 PBMC[2]	-	-	-	-	-	-	-	$<1\times10^{-15}$

P values were calculated using two-sided Fisher's exact tests.

[1]Well-annotated genes in the National Center for Biotechnology Information (NCBI) Reference Sequence database.

[2]Integration sites from previously reported HIV-1-infected human PBMCs[59].

[3]Random integration control (RIC). An *in silico* dataset simulation of random integration sites across the human genome (hg19) were reported previously[59].

Supplemental Table 3. *P* values: Gene density (genes ± 500 kb)

	HIV-8.4kb HeLa	HIV-8.4kb HeLa 10 µM PF74 at 8 hpi	HIV-3.1kb HeLa 10 µM PF74 at 10 hpi	HIV-3.1kb HeLa 100 nM LEN at 10 hpi	HIV-3.1kb HeLa 10 nM LEN at 10 hpi	HIV-3.1kb HeLa	NL4-3 PBMC[1]	RIC[2]
HIV-8.4kb HeLa	-	$<1\times10^{-15}$	$<1\times10^{-15}$	$<1\times10^{-15}$	$<1\times10^{-15}$	$<1\times10^{-15}$	6.04×10^{-1}	$<1\times10^{-15}$
HIV-8.4kb HeLa 10 µM PF74 at 8 hpi	-	-	$<1\times10^{-15}$	$<1\times10^{-15}$	$<1\times10^{-15}$	$<1\times10^{-15}$	$<1\times10^{-15}$	$<1\times10^{-15}$
HIV-3.1kb HeLa 10 µM PF74 at 10 hpi	-	-	-	$<1\times10^{-15}$	$<1\times10^{-15}$	$<1\times10^{-15}$	$<1\times10^{-15}$	$<1\times10^{-15}$
HIV-3.1kb HeLa 100 nM LEN at 10 hpi	-	-	-	-	1.27×10^{-4}	$<1\times10^{-15}$	$<1\times10^{-15}$	$<1\times10^{-15}$
HIV-3.1kb HeLa 10 nM LEN at 10 hpi	-	-	-	-	-	$<1\times10^{-15}$	$<1\times10^{-15}$	$<1\times10^{-15}$
HIV-3.1kb HeLa	-	-	-	-	-	-	$<1\times10^{-15}$	$<1\times10^{-15}$
NL4-3 PBMC[2]	-	-	-	-	-	-	-	$<1\times10^{-15}$

P values were calculated using two-sided Mann-Whitney U tests.

[1]Integration sites from previously reported HIV-1-infected human PBMCs[59].

[2]Random integration control (RIC). An *in silico* dataset simulation of random integration sites across the human genome (hg19) were reported previously[59].

Supplemental Table 4. *P* values: Integration into SPADs

	HIV-8.4kb HeLa	HIV-8.4kb HeLa 10 µM PF74 at 8 hpi	HIV-3.1kb HeLa 10 µM PF74 at 10 hpi	HIV-3.1kb HeLa 100 nM LEN at 10 hpi	HIV-3.1kb HeLa 10 nM LEN at 10 hpi	HIV-3.1kb HeLa	NL4-3 PBMC[1]	RIC[2]
HIV-8.4kb HeLa	-	$<1\times10^{-15}$	$<1\times10^{-15}$	$<1\times10^{-15}$	$<1\times10^{-15}$	7.92×10^{-4}	4.41×10^{-5}	$<1\times10^{-15}$
HIV-8.4kb HeLa 10 µM PF74 at 8 hpi	-	-	$<1\times10^{-15}$	$<1\times10^{-15}$	$<1\times10^{-15}$	$<1\times10^{-15}$	$<1\times10^{-15}$	$<1\times10^{-15}$
HIV-3.1kb HeLa 10 µM PF74 at 10 hpi	-	-	-	$<1\times10^{-15}$	$<1\times10^{-15}$	$<1\times10^{-15}$	$<1\times10^{-15}$	8.24×10^{-1}
HIV-3.1kb HeLa 100 nM LEN at 10 hpi	-	-	-	-	1.37×10^{-3}	$<1\times10^{-15}$	$<1\times10^{-15}$	$<1\times10^{-15}$
HIV-3.1kb HeLa 10 nM LEN at 10 hpi	-	-	-	-	-	$<1\times10^{-15}$	$<1\times10^{-15}$	$<1\times10^{-15}$
HIV-3.1kb HeLa	-	-	-	-	-	-	7.30×10^{-2}	$<1\times10^{-15}$
NL4-3 PBMC[2]	-	-	-	-	-	-	-	$<1\times10^{-15}$

P values were calculated using two-sided Fisher's exact tests.

[1]Integration sites from previously reported HIV-1-infected human PBMCs[59].

[2]Random integration control (RIC). An *in silico* dataset simulation of random integration sites across the human genome (hg19) were reported previously[59].

Supplemental Table 5. *P* values: Integration into LADs

	HIV-8.4kb HeLa	HIV-8.4kb HeLa 10 µM PF74 at 8 hpi	HIV-3.1kb HeLa 10 µM PF74 at 10 hpi	HIV-3.1kb HeLa 100 nM LEN at 10 hpi	HIV-3.1kb HeLa 10 nM LEN at 10 hpi	HIV-3.1kb HeLa	NL4-3 PBMC[1]	RIC[2]
HIV-8.4kb HeLa	-	$<1\times10^{-15}$	$<1\times10^{-15}$	$<1\times10^{-15}$	$<1\times10^{-15}$	7.92×10^{-6}	3.32×10^{-3}	$<1\times10^{-15}$
HIV-8.4kb HeLa 10 µM PF74 at 8 hpi	-	-	1.40×10^{-14}	2.00×10^{-15}	3.92×10^{-9}	2.05×10^{-4}	$<1\times10^{-15}$	$<1\times10^{-15}$
HIV-3.1kb HeLa 10 µM PF74 at 10 hpi	-	-	-	6.77×10^{-1}	3.44×10^{-2}	$<1\times10^{-15}$	$<1\times10^{-15}$	$<1\times10^{-15}$
HIV-3.1kb HeLa 100 nM LEN at 10 hpi	-	-	-	-	6.96×10^{-2}	$<1\times10^{-15}$	$<1\times10^{-15}$	$<1\times10^{-15}$
HIV-3.1kb HeLa 10 nM LEN at 10 hpi	-	-	-	-	-	1.32×10^{-12}	$<1\times10^{-15}$	$<1\times10^{-15}$
HIV-3.1kb HeLa	-	-	-	-	-	-	2.28×10^{-10}	$<1\times10^{-15}$
NL4-3 PBMC[2]	-	-	-	-	-	-	-	$<1\times10^{-15}$

P values were calculated using two-sided Fisher's exact tests.

[1]Integration sites from previously reported HIV-1-infected human PBMCs[59].

[2]Random integration control (RIC). An *in silico* dataset simulation of random integration sites across the human genome (hg19) were reported previously[59].
