# Supplementary material for: HIV-1 uncoating location dictates sites of integration
Source: Nat Commun. 2026 Apr 9;17:4840. doi: 10.1038/s41467-026-71679-3 (PMC13223229; doi:10.1038/s41467-026-71679-3)
Supplement: Supplementary file 2 — Description Of Additional Supplementary File [file 41467_2026_71679_MOESM2_ESM.pdf]

Description of Additional supplementary files

Supplementary Movie 1. Example of a viral core that lost cmHALO and GFP-CA within an NS. Nuclear movement was corrected, and an average projection was generated from the z-slices containing the particle of interest (defined as the z-slice with the highest intensity ± one adjacent z-slice). Time indicates hours:minutes post-infection. Scale bars, 5 µm; 2 µm (insets).

Supplementary Movie 2. Example of a viral core that lost cmHALO within a NS, exited the NS following substantial loss of GFP-CA, and subsequently lost all GFP-CA. Dim GFP-CA signal can be observed outside the NS at 11:50 (hours:minutes) post-infection. Nuclear movement was corrected, and an average projection was generated from the z-slices containing the particle of interest (defined as the z-slice with the highest intensity ± one adjacent z-slice). Time indicates hours:minutes post-infection. Scale bars, 5 µm; 2 µm (insets).

Supplementary Movie 3. Example of a viral core that exited an NS and subsequently lost cmHALO (i.e., ruptured) following PF74 treatment. Nuclear movement was corrected, and an average projection was generated from the z-slices containing the particle of interest (defined as the z-slice with the highest intensity ± one adjacent z-slice). Time indicates minutes relative to PF74 addition. Scale bars, 5 µm; 2 µm (insets).

Supplementary Movie 4. Example of a viral core that lost cmHALO (i.e., ruptured) within an NS and subsequently exited following PF74 treatment. Nuclear movement was corrected, and an average projection was generated from the z-slices containing the particle of interest (defined as the z-slice with the highest intensity ± one adjacent z-slice). Time indicates minutes relative to PF74 addition. Scale bars, 5 µm; 2 µm (insets).

Supplementary Movie 5. Example of a viral core that lost cmHALO (i.e., ruptured) within an NS and subsequently exited following LEN treatment. Nuclear movement was corrected, and an average projection was generated from the z-slices containing the particle of interest (defined as the z-slice with the highest intensity ± one adjacent z-slice). Time indicates minutes relative to LEN addition. Scale bars, 5 µm; 2 µm (insets).

Supplementary Movie 6. Example of SNAP-CPSF6 dissociation from a viral core, which subsequently exited an NS following PF74 treatment. Nuclear movement was corrected, and an average projection was generated from the z-slices containing the particle of interest (defined as the z-slice with the highest intensity ± one adjacent z-slice). Time indicates minutes relative to PF74 addition. Scale bars, 5 µm; 2 µm (insets).

Supplementary Movie 7. Example of SNAP-CPSF6 dissociation from a viral core, which subsequently exited an NS following LEN treatment. Nuclear movement was corrected, and an average projection was generated from the z-slices containing the particle of interest (defined as the z-slice with the highest intensity ± one adjacent z-slice). Time indicates minutes relative to LEN addition. Scale bars, 5 µm; 2 µm (insets).

Supplementary Movie 8. Example of an IN-sfGFP complex exiting an NS following substantial, but not complete, loss of HALO-CA in untreated control cells. Dim HALO-CA signal can be observed outside the NS at 11:00 (hours:minutes) post-infection. Nuclear movement was corrected, and an average projection was generated from the z-slices containing the particle of interest (defined as the z-slice with the highest intensity ± one adjacent z-slice). Time indicates hours:minutes postinfection. Scale bars, 5 µm; 2 µm (insets).

Supplementary Movie 9. Example of an IN-sfGFP complex exiting an NS with minimal loss of HALO-CA following PF74 treatment. Nuclear movement was corrected, and an average projection was generated from the z-slices containing the particle of interest (defined as the z-slice with the highest intensity ± one adjacent z-slice). Time indicates minutes relative to PF74 addition. Scale bars, 5 µm; 2 µm (insets).

Supplementary Movie 10. Example of an IN-sfGFP complex exiting an NS with minimal loss of HALO-CA following LEN treatment. Nuclear movement was corrected, and an average projection was generated from the z-slices containing the particle of interest (defined as the z-slice with the highest intensity ± one adjacent z-slice). Time indicates minutes relative to LEN addition. Scale bars, 5 µm; 2 µm (insets).
